# A heatmap-based deep learning framework for multi-modal registration of VIS, NIR, and thermal images in dermatological imaging

**DOI:** 10.3389/frai.2026.1807482

**Published:** 2026-05-25

**Authors:** Maria Oniga, Paul Florin Rus, Rvazvan Condorovici, Alina Elena Sultana, Andrei Marin, Cristina Cotruţǎ, Daniel Octavian Costache, Horia Blejan, Radu Cristian Jecan, Costinela Corciu

**Affiliations:** 1 Department of Applied Electronics and Information Engineering, National University of Science and Technology POLITEHNICA Bucharest, Bucharest, Romania; 2 Faculty of Medicine, “Carol Davila” University of Medicine and Pharmacy, Bucharest, Romania; 3 Department of Plastic Surgery, St John's Clinical Emergency Hospital, Bucharest, Romania; 4 2nd Dermatology Discipline, Faculty of Medicine, “Carol Davila” University of Medicine and Pharmacy, Bucharest, Romania; 5 Department of Plastic Surgery and Reconstructive Microsurgery, “Carol Davila” University of Medicine and Pharmacy, Bucharest, Romania; 6 Department of Plastic and Reconstructive Microsurgery, Emergency Clinical Hospital, Bucharest, Romania; 7 Clinical Department of Plastic Surgery and Reconstructive Microsurgery, “Prof. Dr. Agrippa Ionescu” Hospital, Bucharest, Romania

**Keywords:** dataset, deep learning, image registration, melanoma, multispectral imaging

## Abstract

**Introduction:**

Multi-modal image registration leverages complementary information from diverse imaging sources to achieve precise spatial alignment. However, aligning visible (VIS), near-infrared (NIR), and thermal (TH) modalities remains challenging due to appearance differences and limited annotated datasets.

**Methods:**

This study proposes a ResU-Net-inspired framework combining heatmap prediction and homography estimation to enable joint NIR-TH registration via shared feature representations. A proprietary dataset of 155 VIS-NIR-TH skin lesion triplets was created from 62 patients, resulting in 465 images in total. Diagnoses were confirmed by dermatologists or histopathology. Lesions were distributed across the face, trunk, and limbs to evaluate registration robustness under spatial variability.

**Results and discussion:**

Experiments showed that NIR-VIS registration consistently outperformed TH-VIS registration, reflecting NIR's richer structural content and higher spectral similarity with VIS. Despite limitations related to dataset size and acquisition variability, the framework demonstrated the feasibility of VIS-NIR-TH triplet registration and provides the first documented dataset of its kind for multi-modal skin lesion imaging.

## 1 Introduction

Recent advancements in computer vision have progressively emphasized the integration of data from several imaging modalities to deliver additional information for intricate analytical tasks. Some examples include video surveillance, where visible (VIS) images are combined with 3D LiDAR or thermal (TH) data for enhanced object detection and environmental monitoring, where VIS and near-infrared (NIR) images are jointly used in remote sensing (Velesaca et al., 2024). In the medical domain, multimodal strategies have been used in fields like radiology, exemplified by the integration of CT, MRI, and ultrasound imaging. In addition, similar techniques have been applied to dermatology, where NIR and TH imaging are combined with conventional VIS imaging to improve the assessment of various skin lesions.

Since employing different imaging modalities requires distinct cameras with varying characteristics, such as focal length and field of view (FOV), a registration step is necessary to ensure that the images correspond to the same spatial scene. Image registration is the process of aligning two or more images of the same scene, acquired at different times, from various viewpoints, or using sensors based on different physical principles, to enable their combination or comparison (Velesaca et al., 2024). In this context, the goal of registration is to spatially transform the moving image so that it matches the reference image, allowing accurate multimodal analysis (Ramadan et al., 2024). The registration process can be broadly categorized into three strategies: area-based, feature-based, and hybrid approaches. Area-based methods align images by directly comparing intensity patterns, while feature-based methods rely on detecting and matching distinctive features to estimate the spatial transformation. Hybrid approaches combine both strategies by integrating region-based information with feature correspondences to compute the transformation (Kuppala et al., 2020).

Over the years, numerous solutions have been proposed, most of which focus on registering images within the same modality. However, with the increasing emphasis on combining multiple modalities for diverse imaging tasks, the registration problem has become considerably more complex and challenging, as corresponding features may appear substantially different due to the distinct physical and spectral characteristics of each source.

Previous works on image registration address both same- and multi-modality scenarios. For same-modality registration, Abdollahinejad and Hashemi (2023) used a Swin Transformer to predict flow matrices with SSIM and homography-based losses, while Liang et al. (2023) proposed a coarse-to-fine transformer with self- and cross-attention and a reprojection consistency loss to refine correspondences. In remote sensing, CNN-based descriptors improve matching robustness under strong appearance variations (Yang et al., 2018), and iterative co-registration methods further enhance pixel-level alignment under challenging conditions (Berton et al., 2024). For multi-modality registration, Wu et al. (2025) introduced MapGlue, combining semantic context and a dual graph-guided mechanism with a large-scale aligned dataset, achieving robust cross-modal matching and generalization.

Image registration is particularly vital in medical imaging, where modeling complex anatomical deformations is essential. Transformer-based architectures have proven effective by capturing both local and global features. For instance, ModeT (Wang et al., 2023) uses multi-head neighborhood attention and competitive weighting to fuse deformation sub-fields, achieving robust results on LPBA and Mindboggle datasets. TransMorph (Chen et al., 2022) combines Transformer and ConvNet components in a hybrid 3D framework, supporting diffeomorphic and Bayesian variants for various registration tasks, while UTR (Qiu et al., 2024) enhances a U-Net encoder with Transformer blocks and global-local attention to capture hierarchical features on LPBA40 and OASIS. Beyond single-modality applications, cross-modal attention strategies have been explored for TRUS-to-MR registration (Song et al., 2022), aligning high-level features through contrastive pre-training. Collectively, these studies highlight a trend toward architectures integrating local precision with global context for intra- and inter-modality registration.

Unlike medical imaging, VIS-NIR registration poses greater challenges due to spectral and appearance differences, which limit structural anchors for alignment. Consequently, robust representation learning has become a focus. Correlation-driven approaches such as CNet (Quan et al., 2022) enhance multimodal feature representations using attention mechanisms and correlation-based loss functions, while RFNet (Xu et al., 2022) jointly optimizes registration and fusion in a mutually reinforcing framework. At the feature-learning level, ReDFeat (Deng and Ma, 2022) couples detection and description using a Super Detector with extended receptive fields to improve cross-spectral matching across VIS, NIR, IR, and SAR domains. More recently, unified architectures like AU-Net (Lu et al., 2025) combine adaptive multi-level fusion with diffusion-based supervision, achieving high performance on large-scale VIS-NIR benchmarks.

VIS-TH registration introduces an even larger modality gap, as TH imagery encodes heat rather than reflected light. To address this, homography-based methods exploit deep feature extraction and attention mechanisms to robustly estimate projective transformations (Debaque et al., 2022), while transformer-based frameworks such as XoFTR leverage masked modeling and coarse-to-fine refinement to achieve precise local correspondences (Tuzcuoglu et al., 2024). Domain-specific applications demonstrate the clinical utility of VIS-TH registration in neurosurgery (Moshaei-Nezhad et al., 2021), combining similarity- and multiresolution-based Demons registration with guided multi-scale fusion to achieve alignment.

Recent trends emphasize domain-invariant frameworks capable of handling multiple modalities. Ren et al. (2025) introduces a unified matching framework by scaling multimodal data generation from RGB inputs, enabling pre-trained matchers such as LightGlue, LoFTR, and RoMa to operate across VIS-NIR, VIS-SAR, and CT-MRI registration. Complementarily, MURF (Xu et al., 2023) integrates registration and fusion in a coarse-to-fine, feedback-driven pipeline for RGB-IR, RGB-NIR, PET-MRI, and CT-MRI, while GRiD (Liu et al., 2024) proposes detector-free, pixel-level matching guided by robust reference points for diverse cross-modal settings. InMIR-Net (Deng et al., 2023) disentangles alignment-relevant and irrelevant features via convolutional sparse coding, enabling interpretable registration across both rigid and non-rigid scenarios.

Despite these advances, most studies focus on pairwise registration, with relatively few addressing triplet alignment. One notable exception is study (Qu et al., 2021), which proposed a Triple-Input Unsupervised Neural Network to register two source images to a common target simultaneously using the LPBA40 MRI dataset. While this work demonstrates the feasibility of triplet registration, its focus is limited to brain MRI scans, restricting broader applicability across modalities and imaging contexts. Therefore, the present study aims to develop a registration pipeline for image triplets across three different modalities: VIS, NIR, and TH. The images were acquired with different cameras, resulting in variations in angle, which necessitate an effective registration process. Thus, we are addressing challenges that prior methods have not fully explored by:

- Developing a pipeline for multi-modal triplet registration across VIS, NIR, and TH images.
- Designing a deep heatmap-based network with attention mechanisms to extract modality-invariant features.
- Introducing a multi-component loss function for structurally coherent keypoint predictions.
- Creating a proprietary dataset of skin lesions (VIS, NIR, and TH images), unique among publicly available datasets.

## 2 Materials and methods

**Figure 1:**
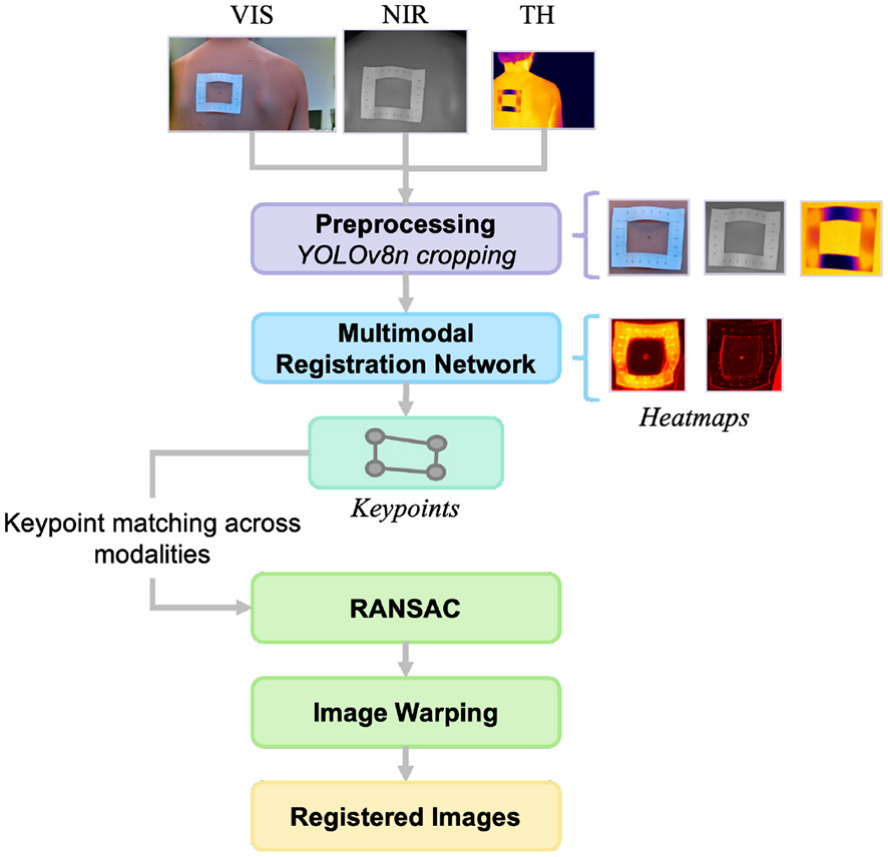
The proposed pipeline for multimodal image triplet registration.

To achieve accurate cross-modal registration and keypoint prediction, we employ a pipeline that combines automated lesion localization and supervised heatmap learning. Each training sample consists of an image triplet and the corresponding keypoints used as pseudo-ground-truth. These keypoints are converted into heatmaps, which are used to train an encoder-decoder network combined with Convolutional Block Attention Modules (CBAM). This methodology ensures robust alignment across modalities while focusing on clinically relevant regions, as presented in Figure 1.

### 2.1 Datasets

#### 2.1.1 Registration dataset

The proposed dataset was acquired over six months in collaboration with Elias Emergency Hospital and St. John's University Hospital, following the protocol and using the imaging system described in Sultana et al. (2025). For acquiring images in the VIS domain, we employed a USB 3.0 camera module that has a 2 MP resolution and utilizes a 1/2“ Sony IMX385 CMOS sensor, coupled with an RGB Marshall Electronics M12-Mount Varifocal Lens. In the NIR case, a Blackfly^®^ S BFS-U3-51S5C-C FLIR camera was used together with a Fujinon HF25SA-1 2/3” C-mount lens, and illumination was provided by LEDs with a peak wavelength of 940 nm. The TH images were acquired with two different cameras from OPTRIS, namely PI450i and PI640i models. The Optris PI 450i model offers sensitivity up to 0.04 K, and has a resolution of 382 × 288, while the PI640i model offers the same sensitivity but has a resolution of 640 × 480. For the VIS case, we obtained an RGB image with a resolution of 1920 × 1080 pixels, while in the NIR case, images are recorded in grayscale at a higher resolution of 2448 × 2048 pixels.

**Figure 2:**
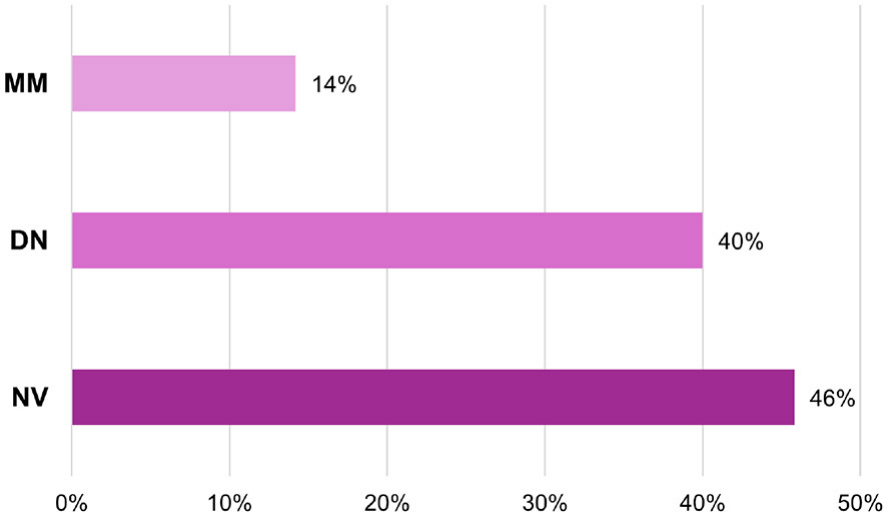
Distribution of annotated cases.

**Figure 3:**
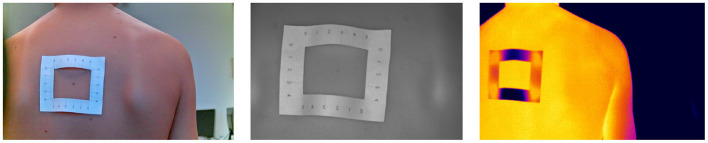
Acquired image triplet: VIS image (**left**), NIR image (**middle**), and TH image (**right**).

The dataset comprises three lesion classes, nevus (NV), dysplastic nevus (DN), and melanoma (MM), with their distribution illustrated in Figure 2. It includes 155 cases from 62 patients, with diagnoses established through expert dermatological assessment or histopathological analysis. For each case, three images were acquired in the VIS, NIR, and TH (long-wavelength infrared, LWIR) spectra, resulting in a total of 465 images. An example of such an image triplet is presented in Figure 3. Lesions occur across diverse anatomical regions, including the face, trunk, and limbs, introducing spatial variability that challenges the robustness of registration algorithms. This heterogeneity in lesion location and morphology provides a rigorous framework for evaluating algorithm performance across different anatomical sites and imaging modalities.

To ensure consistency, each image was assigned a unique identifier comprising the patient number, acquisition date, lesion number, and modality, denoted by the suffixes -v (VIS), -n (NIR), and -t (TH). For instance, 003-180225-1-v corresponds to the VIS image of the first lesion from patient 003, acquired on February 18, 2025. VIS, NIR, and TH images are stored in JPG, BMP, and TIFF formats, respectively, and organized into modality-specific directories. Within each triplet, filenames maintain a common base, with variation only in the modality-specific suffix.

#### 2.1.2 Cropping dataset

**Figure 4:**
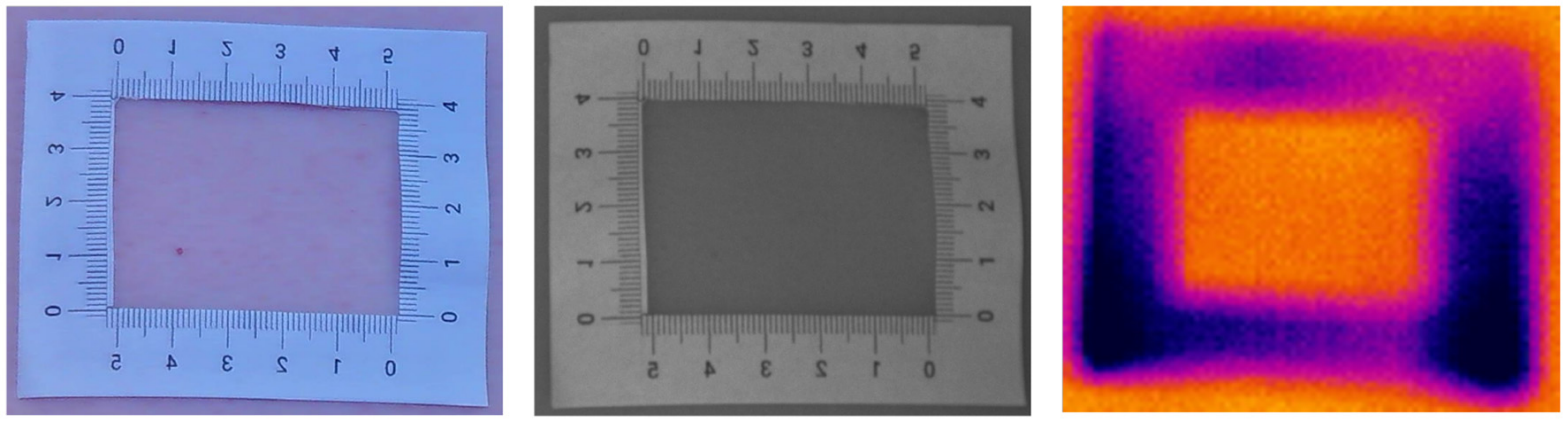
Cropping triplet: VIS image (**left**), NIR image (**middle**), and TH image (**right**).

To facilitate automatic image cropping, an additional dataset was collected under laboratory conditions, specifically designed for the object detection task and distinct from the dataset used for image registration. This dataset comprises 100 triads of VIS, NIR, and TH images, in which the marker was placed on the skin in various positions and orientations, as illustrated in Figure 4. In total, 300 images were manually annotated to localize the squared marker. Annotation was performed using LabelImg (Tzutalin, 2015), producing bounding box coordinates for the marker, which were subsequently used to train the YOLOv8n detector.

#### 2.1.3 Ethics approval and consent to participate

The study was conducted in accordance with the Declaration of Helsinki (1975), as revised in Tokyo in 2004. Ethical approval was obtained from the National University of Science and Technology POLITEHNICA Bucharest, University Ethics Committee, Subcommittee for Research Ethics, approval number 26/06.10.2025. Written informed consent was obtained from all participants before their inclusion in the study, including consent for the acquisition and use of imaging data for research purposes. Participants were informed about the study objectives, procedures, potential risks, and their right to withdraw at any time without consequences.

### 2.2 Preprocessing

To ensure spatial consistency across modalities and focus the registration process on the clinically relevant region, an automated lesion localization step was integrated into the preprocessing pipeline using the YOLOv8n (Varghese and Sambath, 2024) object detection architecture. This model was fine-tuned on a proprietary dataset, presented in Section 2.1.2, with manually annotated bounding boxes around fiducial markers.

The detector was trained to identify a single object class (the marker) under two configurations: (1) modality-specific models for VIS, NIR, and TH domains, and (2) a unified model trained across all modalities to enhance cross-domain robustness. For each modality, the predicted bounding box defined a square crop centered on the lesion, and all resulting patches within each triplet were resampled to a common spatial resolution to ensure correspondence during registration.

### 2.3 Pseudo ground truth generation

Following cropping and resizing, the dataset for cross-modal registration was constructed using keypoint heatmaps. Each training sample consisted of a triplet comprising the fixed VIS image and the corresponding moving NIR and TH images. Given the absence of manually annotated correspondences and the inherent difficulty of obtaining them across modalities, pseudo-ground-truth was generated through an automated procedure.

Specifically, we employed a pre-existing keypoint extraction and matching algorithm (MINIMA-LoFTR) (Ren et al., 2025) to estimate correspondences between the moving and fixed images. Although these automatically derived correspondences do not constitute absolute ground-truth, they provide a strong and reliable approximation of cross-modal landmarks. At the same time, because the supervision signal is derived from a specific matching algorithm, it may introduce a bias toward the keypoint distribution and matching characteristics of MINIMA-LoFTR. In this sense, the pseudo-ground-truth should be interpreted as a supervisory signal rather than an exact reference.

For each image triplet, two independent sets of correspondences were computed: VIS-NIR and VIS-TH. Each set of matched keypoints was converted into a Gaussian heatmap defined in the coordinate system of the VIS image. This resulted in two supervisory target maps per sample, denoted as *H*^*VIS*−*NIR*^ and *H*^*VIS*−*TH*^, which encode the spatial distribution of corresponding points between the VIS image and each moving modality.

During training, although each sample is composed of a VIS-NIR-TH triplet, only the VIS image is provided as direct input to the network. The NIR and TH images are used exclusively to derive the pseudo-ground-truth correspondences and the associated heatmaps. This design avoids explicit early or late fusion of the three modalities and instead formulates the problem as a dual-task prediction in the VIS reference frame.

To maintain consistency, the training and validation stages were conducted exclusively on image triplets, ensuring that the network was continuously exposed to the complete multimodal input configuration. However, it should be noted that the multimodal configuration is reflected in the supervision (target heatmaps) rather than in the network input itself. Importantly, this formulation differs from the original MINIMA-LoFTR pipeline, as the network does not aim to reproduce explicit correspondences but instead learns to predict modality-invariant heatmaps from a single VIS input.

Furthermore, the evaluation metrics used are independent of the pseudo-ground-truth keypoints. This ensures that the reported performance reflects the quality of the final registered images rather than agreement with the MINIMA-LoFTR correspondences. In this context, improvements observed in structural metrics such as SSIM can be attributed to enhanced geometric consistency, indicating that the network is able to regularize and refine the pseudo-ground-truth signal rather than simply replicate it.

### 2.4 Proposed network for multimodal registration

**Figure 5:**
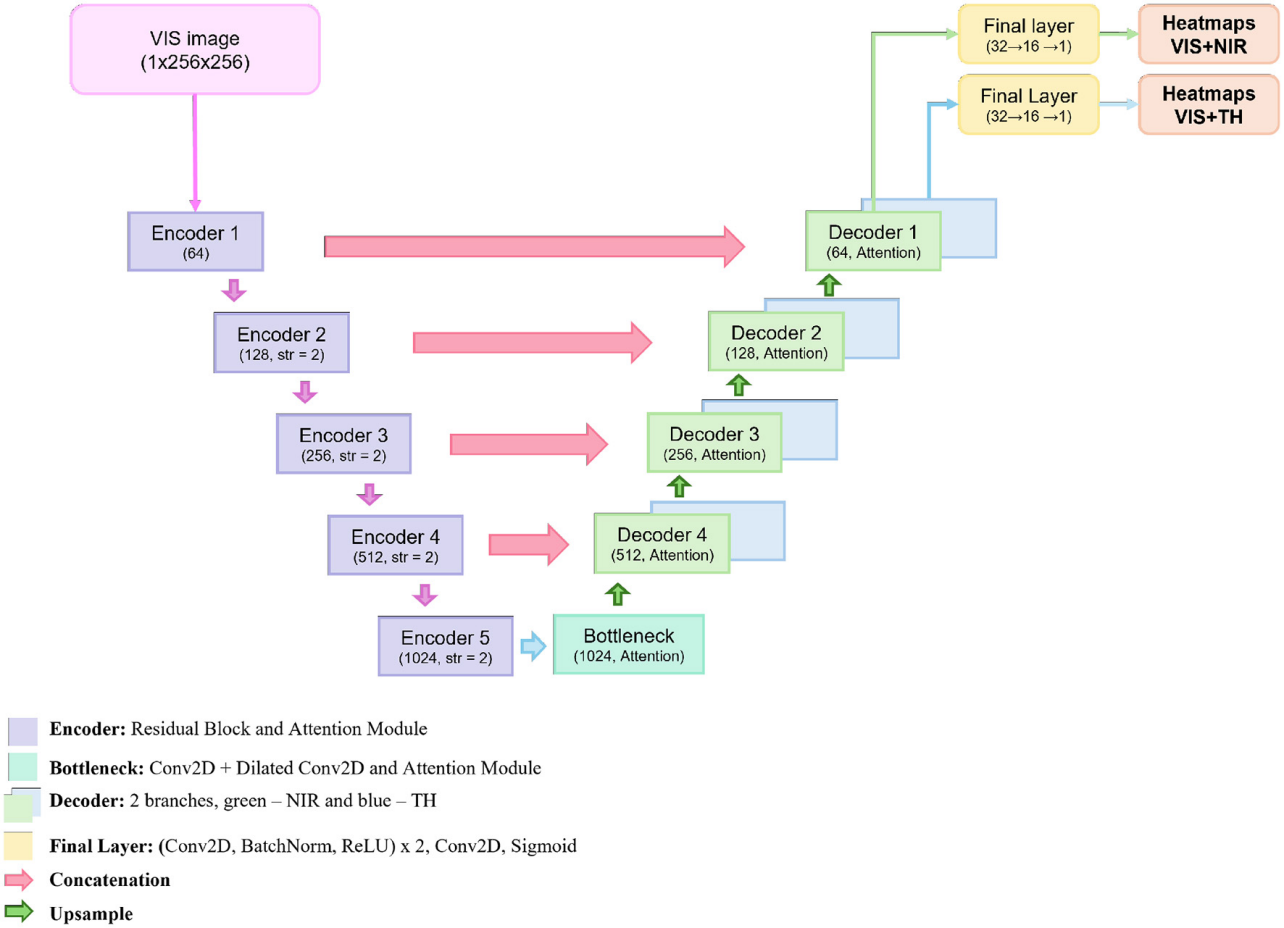
Proposed network architecture.

The proposed framework adopts a deep learning-based strategy for cross-modal registration. Rather than relying on handcrafted descriptors, which often fail under modality-induced appearance changes, our method directly predicts dense keypoint probability maps through a unified heatmap-based network. We introduce a dual-branch network based on the ResU-Net (Diakogiannis et al., 2020) architecture, specifically designed to align moving images from NIR and TH modalities with a fixed VIS reference image, as illustrated in Figure 5. In the implemented model, the network input is the cropped and resized VIS image only, represented as a single-channel grayscale image of size 256 × 256. NIR and TH information is incorporated through separate supervisory target heatmaps derived from automatically estimated VIS-NIR and VIS-TH correspondences.

Cross-spectral registration is challenging due to substantial differences in intensity, contrast, and texture between modalities. To address these challenges, the network incorporates enhanced residual learning with dilated convolutions and spatial-channel attention. Dilated convolutions expand the receptive field while preserving spatial resolution, enabling the network to capture larger structural patterns that remain more consistent across modalities than local intensity variations. Spatial-channel attention further emphasizes these modality-invariant structures by weighting informative regions and feature maps. The shared encoder comprises five Residual Blocks (He et al., 2016), with downsampling being performed via stride-2 convolutions, enabling the network to capture both low-level structural details and high-level semantic information that remain consistent across modalities. More specifically, the encoder stages produce feature maps with 64, 128, 256, 512, and 1024 channels, respectively. The first encoder block preserves the input spatial resolution, whereas the remaining four blocks downsample the feature maps using a stride = 2 in the first convolution of each block. Each residual block contains two 3 × 3 convolutions, each followed by batch normalization, while ReLU activation is applied after the first convolution and after residual addition. A Dropout2d layer with probability 0.1 is introduced after the first convolution. When the number of channels or spatial resolution changes, the shortcut connection is implemented by a 1 × 1 convolution followed by batch normalization.

At the bottleneck and within each decoder stage, CBAM blocks are applied. CBAM sequentially computes channel attention, which adaptively weights the most informative feature channels, and spatial attention, which emphasizes regions critical for alignment. This dual attention mechanism ensures that modality-invariant features and spatially important regions are highlighted, improving the network's ability to generate accurate correspondence maps across spectral domains. The attention module is a spatial-channel attention block: channel attention is computed using both adaptive average pooling and adaptive max pooling, followed by a shared bottleneck formed by two 1 × 1 convolutions with a reduction ratio of 16 and ReLU activation. The resulting channel-attention map is passed through a Sigmoid function and multiplied by the input features. Spatial attention is then computed from the concatenation of the channel-wise average-pooled and max-pooled maps, followed by a 7 × 7 convolution, batch normalization, and Sigmoid activation. This attention module is applied at the end of every encoder residual block, at the bottleneck, and after each decoder refinement block.

The architecture then diverges into two separate decoder branches, one for NIR and one for TH. Each decoder progressively restores spatial resolution using upsampling blocks, integrates encoder features through attention-guided skip connections, and refines feature representations with convolutional blocks. The two decoder branches have identical topology but independent parameters. Their role is to learn two modality-specific prediction tasks from a shared VIS-derived latent representation: one branch predicts the pseudo-ground-truth heatmap associated with VIS-NIR correspondences, while the second branch predicts the pseudo-ground-truth heatmap associated with VIS-TH correspondences. Therefore, the relationship between the two branches is not based on direct feature exchange between NIR and TH, but on a shared encoder and bottleneck followed by task-specific decoding.

Finally, each branch employs a final layer with multi-stage convolutions and residual refinement, followed by a Sigmoid activation to generate modality-specific heatmaps, producing dual outputs that indicate keypoint locations in the NIR and TH images simultaneously. Each decoder stage first upsamples the incoming feature map using a transposed convolution with kernel size 4, stride 2, and padding 1, reducing the number of channels by half. The corresponding encoder skip feature is projected through a 1 **×** 1 convolution, batch normalization, and ReLU activation, after which it is concatenated with the upsampled decoder feature. The fused representation is then refined using two 3 × 3 convolutions with batch normalization and ReLU activation, with Dropout2d (p = 0.1) inserted between the two convolutions. If necessary, bilinear interpolation is applied to match feature-map sizes before concatenation. The final prediction head in each branch consists of a 3 × 3 convolution reducing the channels from 64 to 32, followed by batch normalization and ReLU, then a second 3 × 3 convolution reducing the channels from 32 to 16, again followed by batch normalization and ReLU, and finally a 1 × 1 convolution producing a single-channel output heatmap. A Sigmoid activation constrains the predicted heatmap values to the interval [0, 1].

### 2.5 Loss function

To optimize the registration network, we designed a multi-objective loss function that supervises both NIR and TH branches. The loss integrates complementary terms that enforce pixel-wise similarity, robust keypoint localization, spatial smoothness, cross-modality consistency, and perceptual coherence. The total loss is expressed as:


$$\begin{array}{r} L_{\text{total}}={\text{λ}}_{MSE}\cdot L_{\text{MSE}}+{\text{λ}}_{Focal}\cdot L_{\text{Focal}}+{\text{λ}}_{Cons}\cdot L_{\text{Cons}}\\ +{\text{λ}}_{Cont}\cdot L_{\text{Cont}}+{\text{λ}}_{Perc}\cdot L_{\text{Perc}} \end{array}$$ (1)

Each loss term addresses a specific aspect of registration: MSE enforces global alignment, focal loss refines keypoint localization, spatial consistency preserves smooth structures, contrastive loss regularizes modality differences, and perceptual loss provides feature-level alignment.

To assess the contribution of each loss term, we conduct a systematic ablation study over the composite loss function. Thus, we evaluate two types of modifications: (i) removal of individual loss components by setting their corresponding coefficient to zero, and (ii) variation of each coefficient independently while keeping the remaining terms fixed at their default values. This allows us to isolate the effect of each loss and analyze its sensitivity.

After conducting the experiments, we determined the following weighting coefficients for each loss component:: λ_MSE_ = 0.8, λ_Focal_ = 0.6, λ_Cons_ = 0.4, λ_Cont_ = 0.3, and λ_Perc_ = 0.2. These weights were guided by prior knowledge of the relative importance of each component and yielded stable training behavior and qualitatively satisfactory results.

### 2.6 Homography estimation and image warping

After predicting keypoint heatmaps for each modality, keypoints were extracted using a non-maximum suppression strategy to identify local maxima above a confidence threshold. To improve robustness, a maximum number of keypoints and a suppression radius were applied to avoid overlapping detections.

The extracted keypoints from different modalities were then matched based on spatial proximity, prioritizing pairs with high confidence scores and minimal Euclidean distance. This resulted in a preliminary set of correspondences between the VIS, NIR, and TH modalities.

Using these correspondences, a robust homography was estimated via RANSAC (Liu et al., 2023), allowing for the exclusion of outlier keypoints (Tapu et al., 2015). The resulting homography matrix was validated by checking the determinant of its linear component to prevent degenerate transformations.

Although homography assumes a planar transformation, this approximation is justified in our setting. The region of interest is defined by a small skin patch marked by a rigid square reference, which locally behaves as a quasi-planar surface. In addition, the dominant variations between modalities arise from global patient motion and differences in camera viewpoints and fields of view, which can be effectively modeled by a projective transformation.

Alternative models, such as affine or non-rigid (deformable) registration, were considered. However, affine transformations are insufficient to account for perspective effects introduced by multi-sensor acquisition, while deformable models introduce additional complexity and require dense correspondences or strong regularization. Given that registration is performed with respect to a fixed VIS reference and relies on sparse, high-confidence keypoints, a homography provides a good trade-off between model flexibility and robustness.

Finally, each moving image (NIR or TH) was warped onto the fixed VIS image using the estimated homography. In cases where homography estimation failed, the original image was retained. Warping was performed using a perspective transformation, ensuring that the aligned images shared identical spatial dimensions for subsequent registration analysis.

## 3 Results

### 3.1 Preprocessing

As mentioned earlier, the images in the registration dataset vary in resolution, and the cameras used for acquisition have varying parameters. Consequently, the original images include not only the region of interest (ROI), defined by the squared marker, but also surrounding structures such as additional skin areas. Thus, conventional corner detection proved unsuitable for marker-based cropping. In particular, while the marker is clearly distinguishable with sharp corners in the VIS and NIR modalities, in the TH images, it often appears less visible, and its corners tend to be rounded, which reduces the reliability of corner detection. To overcome this limitation, we employed the YOLOv8n model with a single target class corresponding to the squared marker, conducting two experiments: (1) training separate models for each of the three modalities, and (2) training a single model jointly on all modalities.

**Figure 6:**
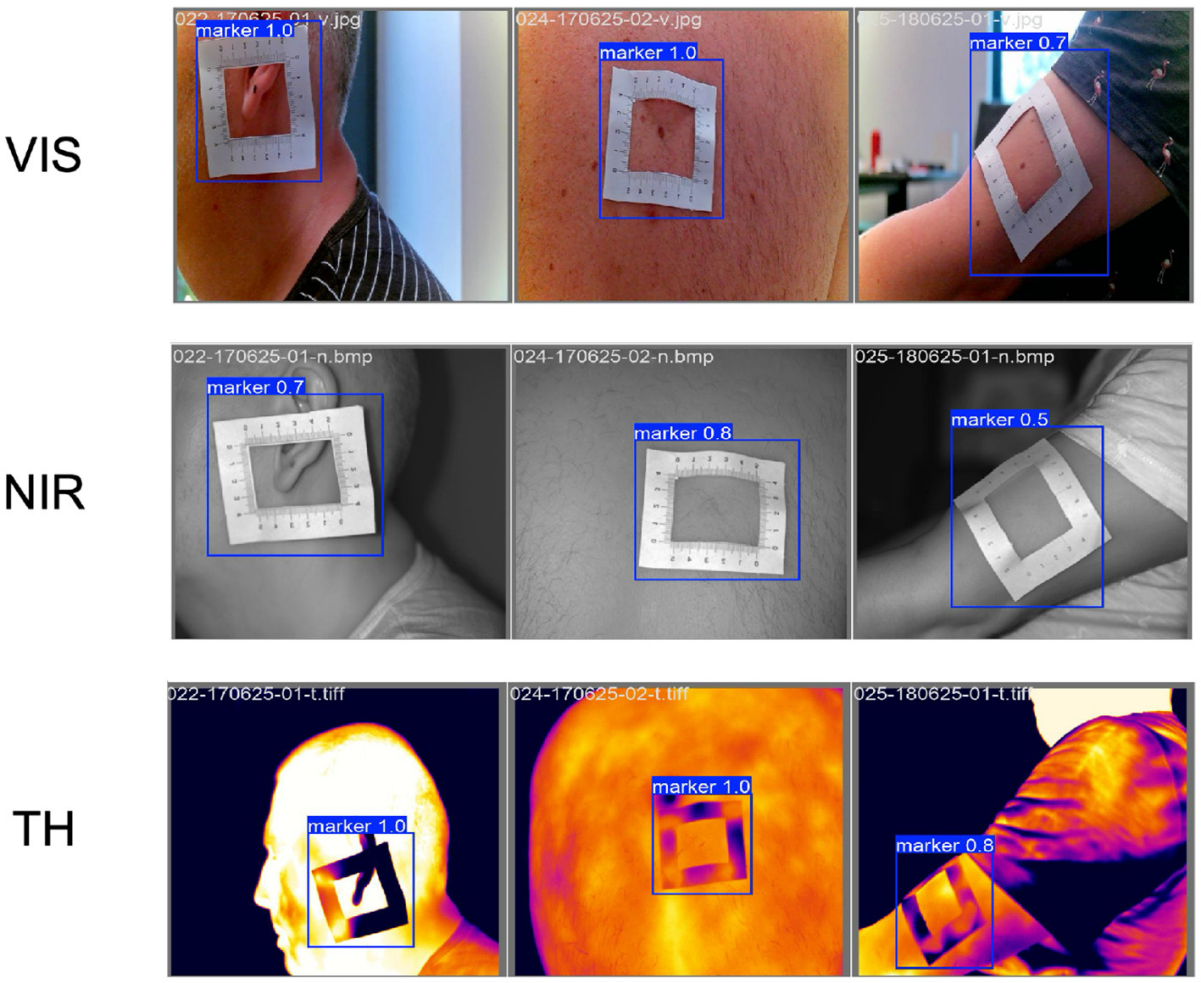
Examples of squared marker detection using YOLOv8n models trained separately for each modality. Rows correspond to the three imaging modalities (VIS, NIR, and TH), and columns illustrate representative detection outcomes.

**Figure 7:**
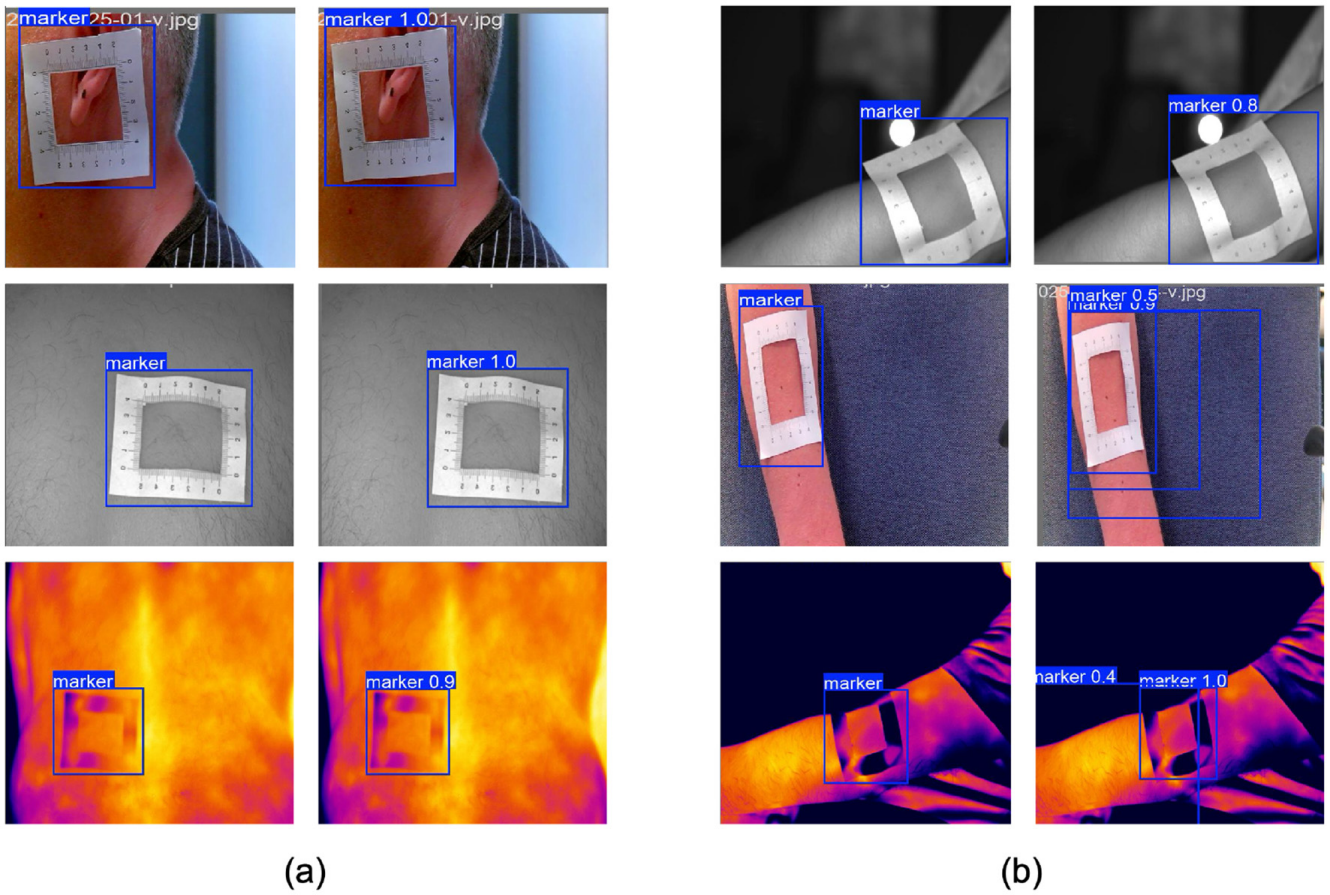
Examples of squared marker detection with YOLOv8n: **(a)** correctly detected markers and **(b)** missed or incorrectly detected markers. In each case, the first column shows the ground-truth label, and the second column shows the prediction from YOLOv8n.

In both experiments described above, the model was initialized with COCO-pretrained weights and trained for 20 epochs with a batch size of 4 and an 80% training and 20% validation split. Training was conducted on a system equipped with an AMD Ryzen 7 4800H CPU (2.90 GHz), 16 GB RAM, and an NVIDIA RTX 3050 GPU with 4 GB VRAM. For both experiments the performance was reported in terms of mAP_50 − 95_, mAP_50_, mAP_75_, mean F1 score, and per-class average precision (AP). The results obtained for the first experiment are presented in Table 1 and Figure 6, and for the second experiment in Table 2 and Figure 7.

**Table 1:** Evaluation metrics obtained during the validation of YOLOv8n models trained separately for each modality.

Modality	mAP_50 − 95_	mAP_50_	mAP_75_	F1	AP
VIS	0.8220	0.9800	0.9063	0.9472	0.8220
NIR	0.8594	0.9950	0.9950	0.9962	0.8594
TH	0.8615	0.9950	0.9950	0.9972	0.8615

**Table 2:** Evaluation metrics obtained during the validation of a single YOLOv8n model trained jointly across all modalities.

mAP50-95	mAP_50_	mAP_75_	F1	AP
0.8489	0.9950	0.9924	0.9838	0.8489

Table 1 presents the performance metrics of YOLOv8n models trained independently for each modality (VIS, NIR, and TH). Across all modalities, the models achieved strong detection performance, as indicated by the consistently high mean Average Precision (mAP) and F1-scores. The VIS model obtained an mAP_50 − 95_ of 0.8220 and an F1-score of 0.9472, reflecting reliable detection performance despite slightly lower precision at stricter overlap thresholds compared to the other modalities. The NIR and TH models demonstrated higher overall accuracy, with mAP_50 − 95_ values of 0.8594 and 0.8615, respectively, and near-perfect F1-scores (0.9962 and 0.9972). Furthermore, mAP_50_ and mAP_75_ were nearly identical and very high for both NIR and TH, confirming that marker detection remained robust under both lenient and strict IoU criteria. These findings indicate that the YOLOv8n architecture effectively adapts to modality-specific variations in marker appearance and offers a reliable solution where conventional corner-based cropping methods are less effective.

As presented in Table 2, the jointly trained model attained a mean Average Precision across IoU thresholds from 0.5 to 0.95 (mAP_50 − 95_) of 0.8489, reflecting strong overall detection performance. The high values of mAP_50_ and mAP_75_ further demonstrate that the majority of markers were correctly localized, even under more stringent overlap requirements. The mean F1-score corroborates the model's ability to consistently identify markers across variations in orientation, position, and partial visibility. Nonetheless, several limitations were observed. In some cases, the model produced duplicate detections of a single marker, generated bounding boxes with reduced localization accuracy, or failed to detect the marker entirely. Representative examples of successful detections alongside negative cases are provided in Figure 7.

After extracting the coordinates of the predicted bounding box around the squared marker, cropping was performed to isolate the region of interest. While training separate YOLOv8n models for each modality yielded high detection accuracy, the jointly trained model achieved comparable performance and provided the practical advantage of a single, unified detector. This strategy facilitated consistent marker localization across modalities and streamlined the cropping process by eliminating the need to maintain multiple models. However, manual adjustment was required in 4 TH cases, corresponding to approximately 3.4% of the 155 image triplets, where the predicted bounding box did not fully encompass the marker. Therefore, the preprocessing stage should be regarded as semi-automated rather than fully automated. For accurate registration, all images were standardized to a common resolution.

### 3.2 Registration

To perform the registration process, we proposed a ResU-Net-like architecture that can successfully predict heatmaps, from which keypoints are extracted. The latter are used in homography matrix estimation and, ultimately, in the image warping process. To train our model, we used a proprietary dataset, described in Section 2.1.1, where keypoints were automatically extracted using the MINIMA-LoFTR method (Ren et al., 2025) and treated as pseudo-ground-truth to supervise the network.

**Figure 8:**
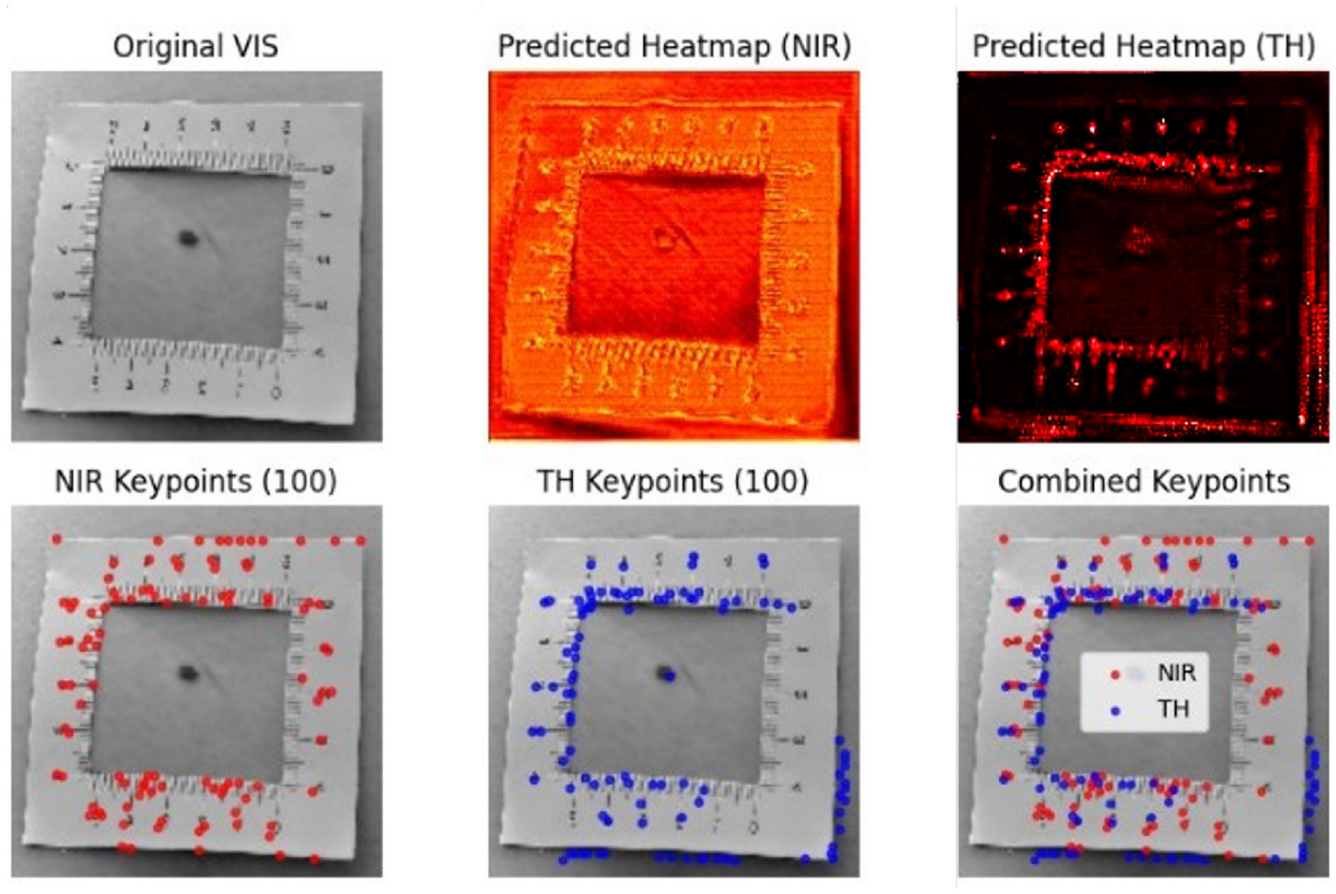
Comparison of predicted and ground truth heatmaps for image registration.

From the obtained keypoints, we generated heatmaps, which were further used in the training process, as seen in Figure 8. The extracted keypoints are primarily located on the squared marker edges and corners, ensuring good spatial correspondence across modalities, and the predictions capture the essential patterns and provide sufficient constraints for cross-modal registration. In all cases, we ensured that the training and validation datasets contained only image triplets.

The training process was conducted on a system equipped with an AMD Ryzen 7 5700G CPU (2.90 GHz), 16 GB RAM, and an NVIDIA RTX 3060 GPU with 12 GB VRAM. The network was implemented in PyTorch and trained using the AdamW optimizer with gradient clipping (maximum norm of 1.0). The dataset was split into train and validation subsets at the patient level, such that data from the same patient were not allowed to appear in both subsets, allowing a more reliable assessment of generalization. Using an 80/20 patient-level split, 49 patients were assigned to the training subset and 13 patients to the validation subset, resulting in 126 training triplets and 29 validation triplets.

During training and validation, the VIS images were converted to grayscale and resized to 256 × 256 pixels. Since the dataset is relatively small, a data augmentation process was employed for the training images, which included: random rotations, color jitter, and random affine transformations such as translation and scaling. In addition, to avoid overfitting, the network incorporates explicit regularization through Dropout2d layers in both the encoder-decoder blocks and the bottleneck, while AdamW introduces weight decay during optimization. The model used for evaluation was selected according to validation loss, rather than training loss, in order to reduce the risk of an overfitting solution.

Hyperparameters such as the learning rate and number of training epochs were determined through preliminary experiments, while the batch size was fixed at 8. Model performance was evaluated using SSIM (Structural Similarity Index Measure), MI (Mutual Information), and NMI (Normalized Mutual Information), which respectively quantify structural alignment, statistical dependency, and normalized shared information between the reference and registered images.

**Figure 9:**
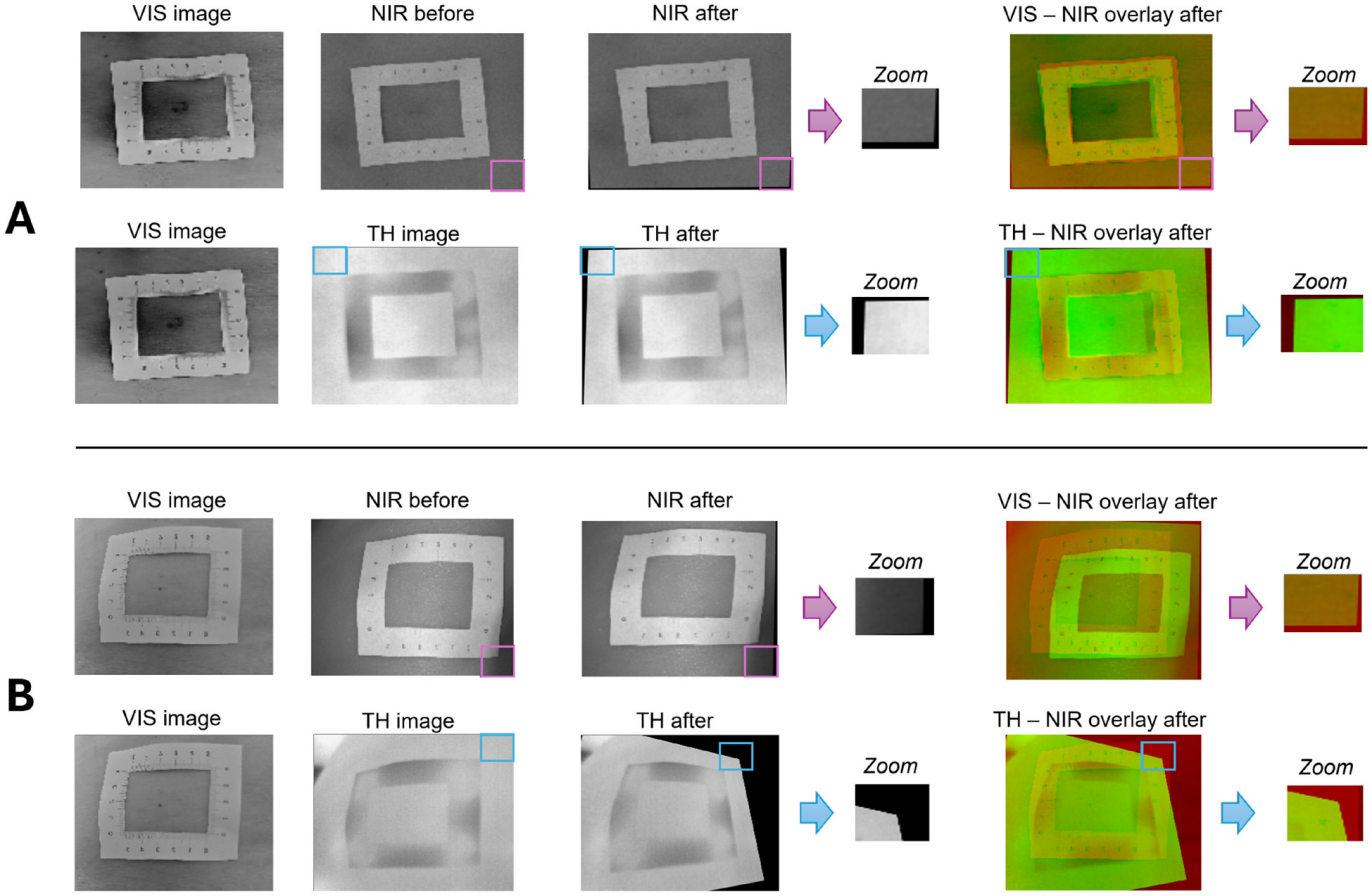
Comparison of multimodal registration results. **(A)** Favorable case and **(B)** challenging case. For each scenario, we show the reference VIS image, the input NIR/TH image before registration, and the aligned result after applying the proposed method. Difference maps highlight the reduction in pixel-wise discrepancies. Zoomed regions emphasize improvements in local alignment, particularly along marker boundaries.

To assess the stability and convergence of the proposed approach, we conducted experiments varying the learning rate and the number of training epochs. Based on the results in Table 3, a learning rate of 1 × 10^−5^ and 30 training epochs yielded the best performance across the SSIM, MI, and NMI metrics. NIR-VIS registration consistently outperformed TH-VIS registration, achieving higher SSIM values and stronger MI and NMI correlations, due to closer spectral and textural characteristics. In contrast, TH-VIS registration proved more challenging, showing greater variability and lower metric values, reflecting the substantial spectral gap and reduced contrast in TH imagery. Representative examples of successful and suboptimal alignments are illustrated in Figure 9.

**Table 3:** The effect of varying epochs and learning rates on NIR-VIS and TH-VIS registration metrics.

Epochs	LR	SSIM		MI		NMI	
		NIR	TH	NIR	TH	NIR	TH
20	1e-4	0.458	0.338	0.997	0.673	0.147	0.103
20	1e-5	0.483	0.312	1.010	0.634	0.147	0.100
20	5e-4	0.481	0.310	0.993	0.636	0.152	0.099
20	5e-5	0.490	0.302	1.012	0.588	0.151	0.093
25	1e-4	0.488	0.380	0.982	0.560	0.148	0.086
25	1e-5	0.490	0.376	1.005	0.661	0.149	0.104
**30**	**1e-5**	**0.496**	**0.395**	**1.073**	**0.732**	**0.154**	**0.112**

Bold values indicate the best performance obtained across the evaluated hyperparameter configurations.

To analyze the influence of the loss components and their relative weighting, we conducted an ablation study, the results of which are reported in Table 4. The study shows that the final configuration provides the most balanced performance across all metrics and modalities. While certain configurations yield improvements in individual metrics (e.g., higher TH SSIM or MI), these gains are not consistent and often come at the expense of performance in other modalities or metrics. In particular, removing the focal loss leads to a substantial degradation, highlighting its critical role in guiding accurate keypoint localization. Similarly, extreme weighting of individual loss terms results in unstable behavior, especially for TH-VIS registration. Based on these observations, the full configuration was selected for all subsequent experiments, as it ensures stable and consistent performance across both structural and information-theoretic measures.

**Table 4:** Ablation study of the proposed loss formulation.

Setting	λ_*mse*_	λ_*focal*_	λ_*cons*_	λ_*cont*_	λ_*perc*_	NIR-SSIM	NIR-MI	NIR-NMI	TH-SSIM	TH-MI	TH-NMI
*Final version*	*0.8*	*0.6*	*0.4*	*0.3*	*0.2*	*0.4648*	*0.9951*	*0.1464*	*0.3211*	*0.6834*	*0.1063*
Mo MSE	0.0	0.6	0.4	0.3	0.2	0.4649	1.0158	0.1482	0.3198	0.6788	0.1065
No focal	0.8	0.0	0.4	0.3	0.2	0.3715	0.7524	0.1107	0.3318	0.6716	0.1038
No consistency	0.8	0.6	0.0	0.3	0.2	0.4746	1.0056	0.1462	0.3681	0.7127	0.1099
No contrastive	0.8	0.6	0.4	0.0	0.2	0.4716	1.0116	0.1470	0.3051	0.7100	0.1097
No perceptual	0.8	0.6	0.4	0.3	0.0	0.4714	1.0049	0.1462	0.3442	0.7142	0.1109
Low MSE	0.2	0.6	0.4	0.3	0.2	0.4530	1.0055	0.1474	0.3505	0.6478	0.0991
Medium MSE	0.4	0.6	0.4	0.3	0.2	0.4714	1.0157	0.1476	0.3608	0.6632	0.1023
High MSE	1.2	0.6	0.4	0.3	0.2	0.4514	1.0036	0.1472	0.3043	0.6159	0.0963
Low focal	0.8	0.1	0.4	0.3	0.2	0.4321	0.8827	0.1332	0.2660	0.5990	0.0927
Medium focal	0.8	0.3	0.4	0.3	0.2	0.4638	1.0114	0.1474	0.3948	0.7919	0.1193
High focal	0.8	0.9	0.4	0.3	0.2	0.4542	0.9946	0.1458	0.2559	0.5895	0.0943
Low consistency	0.8	0.6	0.2	0.3	0.2	0.4669	1.0077	0.1467	0.3771	0.7409	0.1145
Medium consistency	0.8	0.6	0.6	0.3	0.2	0.4702	1.0077	0.1473	0.3398	0.6268	0.0976
High consistency	0.8	0.6	0.8	0.3	0.2	0.4493	0.9430	0.1406	0.2156	0.5165	0.0829
Low contrastive	0.8	0.6	0.4	0.15	0.2	0.4524	0.9821	0.1445	0.2695	0.5148	0.0823
Medium contrastive	0.8	0.6	0.4	0.45	0.2	0.4688	1.0014	0.1458	0.2520	0.5420	0.0862
High contrastive	0.8	0.6	0.4	0.6	0.2	0.4735	1.0043	0.1463	0.2080	0.5240	0.0825
Low perceptual	0.8	0.6	0.4	0.3	0.1	0.4652	1.0003	0.1466	0.2876	0.6450	0.0998
Medium perceptual	0.8	0.6	0.4	0.3	0.3	0.4517	1.0064	0.1486	0.3665	0.7385	0.1129
High perceptual	0.8	0.6	0.4	0.3	0.4	0.4668	1.0074	0.1465	0.3007	0.6537	0.1028

Each configuration adjusts a single loss coefficient relative to the default setting.

**Figure 10:**
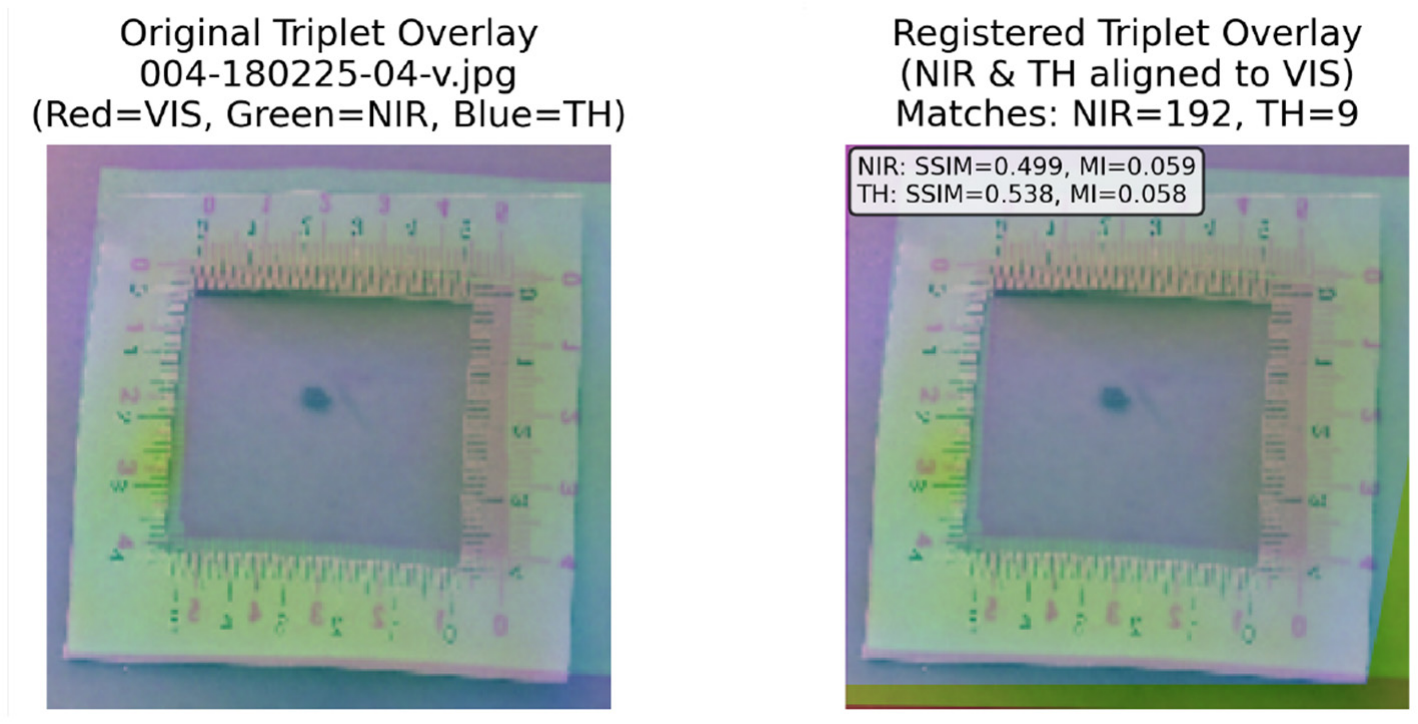
Comparison between the overlay of the original images and the overlay of the registered images.

Qualitative evaluation of the multispectral registration results revealed clear differences between modalities, as seen in Figure 10. NIR-based registration consistently achieved higher keypoint detection counts, with 164, 192, and 181 matches, compared to 44, 9, and 23 matches for TH registration. The registered image pairs demonstrated superior alignment quality for the NIR modality, clearly observed in structured anatomical features and geometric markers, while TH registration exhibited increased difficulty, likely attributable to reduced feature contrast.

To further assess the performance of the proposed method, we also employed the solutions presented in DeepConvFeatures (Yang et al., 2018), VISMATCH (Berton et al., 2024), and MINIMA-LoFTR (Ren et al., 2025) on our dataset and computed the same metrics for the registered data, namely SSIM, MI, and NMI. VISMATCH and DeepConvFeatures are two classical registration approaches, employed to provide a broader comparison against both learning-based and traditional methods. All reported values correspond to the mean across image pairs and are accompanied by standard deviation. It should also be noted that the number of evaluated triplets differs across methods (Proprietary method, DeepConvFeatures, and MINIMA: 29, and VISMATCH: 7), which is taken into account in the statistical analysis. The results are summarized in Table 5.

**Table 5:** Comparison of registration performance across methods.

Method	NIR			TH		
	SSIM	MI	NMI	SSIM	MI	NMI
DeepConvFeatures (Yang et al., 2018)	0.202 ± 0.080	0.700 ± 0.187	0.160 ± 0.035	0.123 ± 0.062	0.460 ± 0.138	0.118 ± 0.030
MINIMA (Ren et al., 2025)	0.440 ± 0.096	0.936 ± 0.191	**0.202** ± 0.035	0.209 ± 0.073	0.683 ± 0.211	**0.154** ± 0.038
VISMATCH (Berton et al., 2024)	0.213 ± 0.086	0.626 ± 0.205	0.141 ± 0.034	0.083 ± 0.116	0.240 ± 0.106	0.073 ± 0.029
**Proprietary**	**0.496** ± 0.110^*^	**1.073** ± 0.283^*^	0.154 ± 0.036	**0.395** ± 0.180^*^	**0.732** ± 0.306^*^	0.112 ± 0.044

Values are reported as mean ± standard deviation. ^*^indicates statistically significant improvement over other methods (*p* < 0.05, Welch's *t*-test). Best results for each metric are shown in bold.

Table 5 presents a direct comparison between the MINIMA-LoFTR, VISMATCH, DeepConvFeatures solutions, and our proprietary model applied to the same dataset. Overall, VISMATCH and DeepConvFeatures consistently underperformed across all evaluated metrics, particularly in the TH modality, whereas MINIMA-LoFTR and our method achieved higher and more competitive results. In terms of SSIM, our model achieves the highest values for both NIR (0.496) and TH (0.395) images, outperforming MINIMA-LoFTR (0.440 and 0.209, respectively), VISMATCH (0.213 and 0.083), and DeepConvFeatures (0.202 and 0.123). The improvement is especially pronounced in the TH modality, where the difference is substantial, highlighting the ability of the proposed method to better preserve structural alignment in challenging LWIR conditions. A similar trend is observed for MI, where our method achieves the highest values for both NIR (1.073) and TH (0.732), exceeding MINIMA-LoFTR (0.936 and 0.683) and clearly surpassing VISMATCH (0.626 and 0.240) and DeepConvFeatures (0.700 and 0.460). This indicates improved statistical dependency between aligned images. Overall, these results demonstrate that the proposed model improves both structural and information-theoretic metrics, particularly for TH images, while maintaining a consistent advantage over classical baselines and competitive performance with state-of-the-art learning-based approaches.

Statistical analysis using Welch's *t*-test further confirms that the improvements of our method over VISMATCH and DeepConvFeatures are significant (*p* < 0.05) for SSIM and MI, as indicated in Table 5, while differences with MINIMA-LoFTR are not statistically significant for these metrics. These findings highlight current limitations, and future work will focus on refining the registration process to close the performance gap with MINIMA-LoFTR while maintaining the advantage of simultaneous triplet alignment. The evaluation metrics (SSIM, MI, NMI) are independent of the pseudo-ground-truth used during training, ensuring that the results reflect true registration performance rather than overfitting to MINIMA-LoFTR keypoints.

To further assess the variability of the results, 95% confidence intervals were computed for all methods and metrics. The intervals confirm the trends observed in Table 6, with our method showing consistently higher and well-separated ranges compared to VISMATCH and DeepConvFeatures, particularly for SSIM and MI in both modalities. In contrast, partial overlap with MINIMA-LoFTR is observed for certain metrics, indicating comparable performance in these cases. Overall, the confidence intervals support the statistical analysis, highlighting stable improvements over classical baselines while confirming competitive behavior with state-of-the-art methods.

**Table 6:** 95% confidence intervals for all evaluated metrics and methods.

Method	NIR (95% CI)			TH (95% CI)		
	SSIM	MI	NMI	SSIM	MI	NMI
DeepConvFeatures (Yang et al., 2018)	[0.173, 0.231]	[0.632, 0.768]	[0.147, 0.173]	[0.100, 0.146]	[0.410, 0.510]	[0.107, 0.129]
MINIMA (Ren et al., 2025)	[0.405, 0.475]	[0.866, 1.006]	[0.189, 0.215]	[0.182, 0.236]	[0.606, 0.760]	[0.140, 0.168]
VISMATCH (Berton et al., 2024)	[0.149, 0.277]	[0.474, 0.778]	[0.116, 0.166]	[-0.003, 0.169]	[0.161, 0.319]	[0.051, 0.095]
**Proprietary**	[0.453, 0.539]	[0.962, 1.184]	[0.140, 0.168]	[0.325, 0.465]	[0.612, 0.852]	[0.095, 0.129]

**Figure 11:**
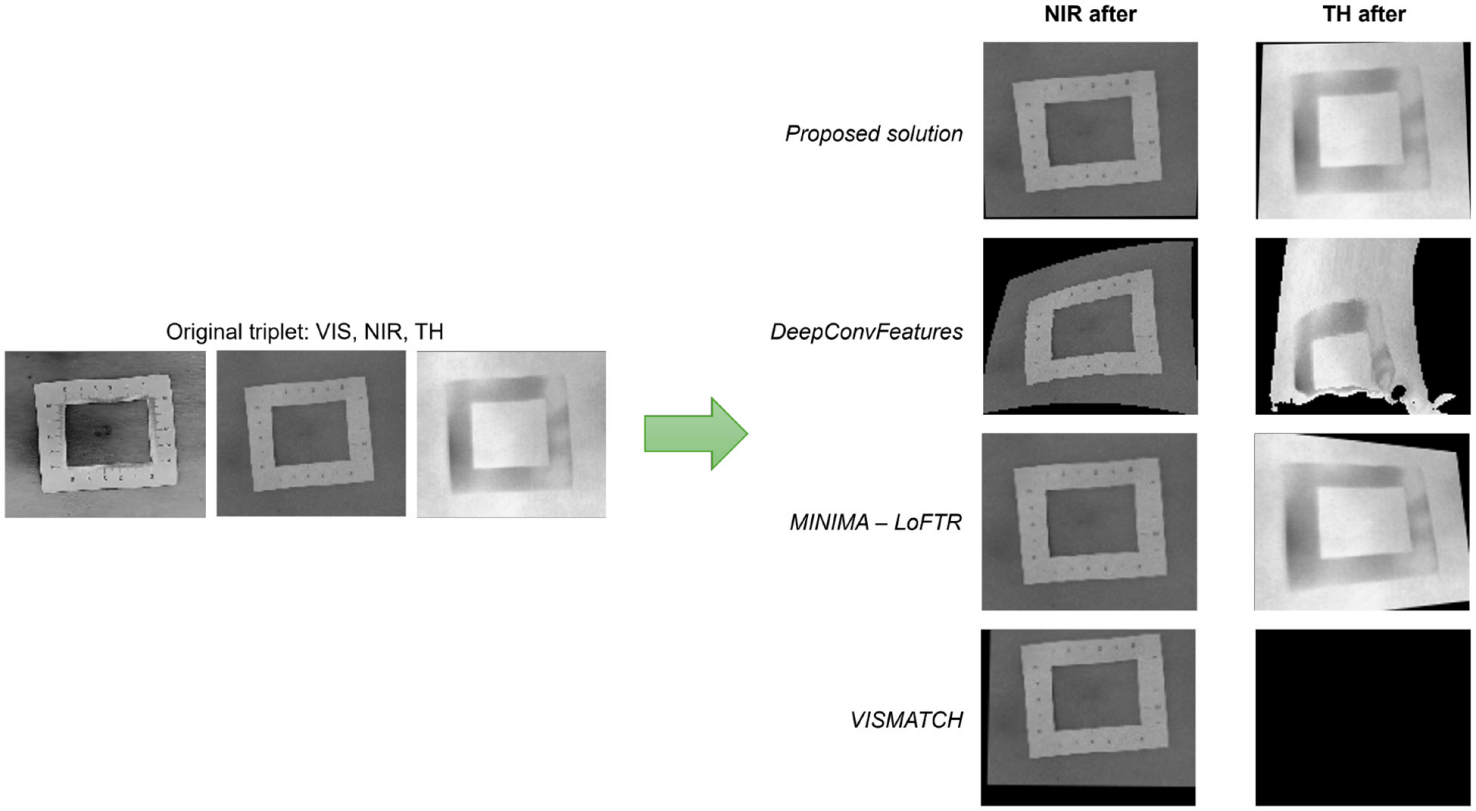
Qualitative comparison of multi-modal registration results.

In addition, Figure 11 provides a qualitative comparison between the original triplets, and the corresponding registration results obtained using our proposed method, MINIMA-LoFTR, VISMATCH, and DeepConvFeatures. The original images exhibit noticeable spatial misalignment between the VIS and TH modalities, particularly in the positioning of the lesion and the square marker. After registration with our model, a clear improvement in alignment is observed, particularly for the TH image. Thus, the lesion and the marker boundaries are better matched across modalities, preserving the overall spatial coherence. By contrast, VISMATCH and DeepConvFeatures often fail to achieve meaningful alignment and may introduce noticeable geometric distortions. MINIMA-LoFTR produces partial corrections and improves alignment in some regions, but inconsistencies and residual misalignment remain, especially in the TH modality. These visual results reinforce the quantitative findings, showing that our method more effectively preserves structural correspondence for VIS-TH registrations.

## 4 Discussions

This study introduces several key contributions in the field of multimodal skin image registration. First, we developed a pipeline capable of functioning on a highly challenging dataset, particularly with respect to the TH modality, where lesions are often faint or absent. Despite these limitations, the network demonstrates the feasibility of simultaneously aligning VIS, NIR, and TH images, even when one or more modalities exhibit severe geometric distortions. The architecture incorporates two decoder branches that account for modality-specific characteristics while leveraging shared representations, enabling coherent triplet registration, which is not possible with conventional pairwise methods.

Another important contribution is the dataset itself. To the best of our knowledge, this is the first dataset including three imaging modalities (VIS, NIR, TH) for skin lesion assessment. Existing datasets typically focus on either VIS or VIS-NIR, and thus, our tri-modal dataset provides a unique resource for advancing cross-modal registration research. Additionally, two complementary strategies for automatic cropping of the region of interest were implemented, ensuring consistent preprocessing despite variability in image acquisition.

The training and validation process also incorporated careful compromises. Hyperparameters (learning rate and number of epochs) were optimized in a cascaded manner, and keypoint extraction was balanced across modalities: NIR images, being visually closer to VIS, contained more detectable keypoints than TH images, so up to 100 keypoints per image were enforced to ensure consistency. These design decisions ensured fair evaluation and reproducibility.

Beyond static images, the proposed framework is extensible to video sequences. To ensure temporal consistency, consecutive frames can be registered to a common reference frame (e.g., frame *t*), allowing the predicted heatmaps at time *t*+1 to be geometrically aligned to frame *t* using the estimated transformation. A temporal consistency constraint can then be enforced between the aligned heatmaps to maintain stable keypoint localization across frames. Once refined, the pipeline can operate on sequences longer than three frames, maintaining consistent multimodal alignment over time, a promising direction for clinical applications where temporal consistency is critical.

Despite these contributions, multi-modal registration remains challenging. TH imaging introduced the most significant difficulties, as lesions were often not visible, removing one of the key anatomical landmarks required for matching. Furthermore, while markers were clear in VIS and NIR, TH images captured only a simplified geometric shape without detailed structural information. Furthermore, difficulties arose from the multi-camera acquisition system. Despite preprocessing steps such as cropping and resizing, the use of three cameras with different specifications introduced variability in the dataset. These factors caused significant variability in orientation, viewing angles, and magnification levels, creating scenarios where identical physical scenes appeared different across modalities. The use of evolving hardware configurations during data collection compounded the problem, generating inconsistencies that limited the effectiveness of conventional registration methods.

The limited dataset size (155 image triplets) posed a challenge, constraining the training process and potentially limiting the model's ability to perform robustly on unseen data. TH images, in particular, exhibited inconsistencies across triplets, which affected feature stability and reduced the effectiveness of conventional registration methods. To mitigate overfitting, the proposed training strategy combines data augmentation, dropout-based regularization, weight decay, and validation-based model selection. Furthermore, the dataset was split at the patient level, preventing leakage of patient-specific characteristics between the training and validation subsets. This aspect is particularly important in dermatological imaging, where skin texture, lesion morphology, and acquisition conditions may be correlated within the same patient, potentially leading to overly optimistic performance estimates if not properly controlled. Nevertheless, expanding the dataset with additional cases and increased variability in acquisition conditions would further improve the robustness and generalization of the proposed approach.

Our network was trained using pseudo-ground-truth keypoints generated with MINIMA-LoFTR (Ren et al., 2025). Although these keypoints provide reliable supervision, the evaluation metrics reported in Table 5 (SSIM, MI, NMI) are independent of the pseudo-ground-truth, ensuring that the observed performance reflects genuine cross-modal registration capability rather than agreement with the MINIMA-LoFTR correspondences. Across all evaluated methods, the proposed approach consistently outperforms classical baselines, namely VISMATCH and DeepConvFeatures, with substantial improvements observed for both SSIM and MI in NIR and TH modalities. These gains indicate enhanced structural alignment and stronger statistical dependency between modalities. When compared to MINIMA-LoFTR, our method achieves higher SSIM and MI values for both modalities, while MINIMA-LoFTR remains competitive in terms of NMI. This suggests that MINIMA-LoFTR may better optimize certain information-theoretic criteria, whereas the proposed model improves geometric consistency and spatial coherence, particularly in challenging TH-VIS scenarios. Overall, these results indicate that the network does not simply reproduce the pseudo-ground-truth correspondences but instead learns a more robust and modality-invariant representation, leading to improved alignment performance across both classical and learning-based baselines.

Overall, this work provides a promising foundation for tri-modal registration, demonstrating feasibility on a highly challenging dataset, introducing the first VIS-NIR-TH skin lesion dataset, and proposing methodological improvements such as triplet registration and robust cropping strategies.

## 5 Conclusion

In this study, we presented a comprehensive approach for multi-modal registration of VIS, NIR, and TH images in dermatological imaging, addressing challenges that have hindered prior methods. Our ResU-Net-like architecture predicts heatmaps for keypoint extraction and simultaneously registers NIR and TH images using dual decoder branches. Despite the limited size and variability of our proprietary dataset, the method demonstrated robust performance, with NIR-VIS registration consistently outperforming TH-VIS across multiple metrics, reflecting the greater spectral similarity and richer feature content in NIR images.

The proprietary dataset itself is a key contribution, providing triplets across three modalities for skin lesions, currently unavailable in publicly accessible datasets, and serving as a valuable resource for future research in multi-modal dermatological imaging. Although the registration results are not as high as might be expected, they establish a solid foundation for future studies, offering opportunities to refine the method and achieve improved alignment performance in challenging multi-modal scenarios. Future work will focus on enhancing the current pipeline to achieve more accurate registration, improving the loss function to better guide multimodal alignment, and expanding the dataset to enable improved generalization and a more robust approach. In addition, the method will be extended to dynamic scenarios, enabling alignment of frames in video sequences and facilitating consistent multimodal registration over time.

## Data Availability

The raw data supporting the conclusions of this article will be made available by the authors, without undue reservation.
